# Not all weight loss is psychiatric: Diagnostic bias in pediatric Crohn's disease

**DOI:** 10.1002/jpr3.70187

**Published:** 2026-04-16

**Authors:** Karolina Aleksandra Jamrozik, Martina Begucci, Vanessa Nadia Dargenio, Nathali Bergmann, Lea Charlotte Hellberg, Hannah Franziska Schumacher, Fernanda Cristofori, Ruggiero Francavilla

**Affiliations:** ^1^ Faculty of Medicine University of Warmia and Mazury in Olsztyn Olsztyn Poland; ^2^ Pediatric Section ‐ Interdiscipilary Department of Medicine University of Bari Bari Italy; ^3^ Faculty of Medicine University of Rostock Rostock Germany

**Keywords:** anorexia nervosa, diagnostic delay, inflammatory bowel disease

## Abstract

Crohn's disease (CD) is a chronic inflammatory bowel disease (IBD) that can often be misdiagnosed, particularly in the female population. This is especially true in the initial stages, which are frequently characterized by weight loss and, at times, dietary intake restriction. We present the case of a patient who experienced a misdiagnosis of anorexia nervosa (AN), leading to significant diagnostic delay (DD) of approximately 8 months and failure to initiate timely treatment. Greater awareness among clinicians and the early utilization of non‐invasive markers, such as fecal calprotectin, could significantly aid in achieving an accurate diagnosis right from the disease's onset.

## 1 INTRODUCTION

Crohn's disease (CD) is an inflammatory bowel disease (IBD) characterized by segmental, transmural inflammation of the gastrointestinal tract. Although CD is typically diagnosed in early adulthood, in approximately 10% of patients, CD occurs during childhood or adolescence, in whom it may cause growth failure, delayed puberty, and significant weight loss.^1^

CD may present with symptoms that resemble those of anorexia nervosa (AN), including weight loss and reduced appetite. This clinical overlap complicates the differential diagnosis, as both conditions can result in malnutrition and gastrointestinal symptoms such as abdominal pain and diarrhea, which may further reduce food intake.^2^, ^3^

Accurate differentiation is crucial, as misdiagnosis or diagnostic delay (DD) can lead to inappropriate management and increased risk of complications.^1^

This report describes the case of a 15‐year‐old girl with CD whose presentation, characterized by weight loss, amenorrhea, and emotional distress, was initially misinterpreted as AN, leading to a considerable DD.

## 2 CASE REPORT

A 15‐year‐old girl was admitted to the Emergency Department for progressive weight loss (>20 kg) over 8 months, which had worsened in the preceding 3 weeks.

Family, perinatal, and developmental history were unremarkable. Her past medical history was non‐contributory.

Six months before admission, she had been evaluated by her general practitioner for fatigue, loss of appetite, and weight loss. She reported emotional distress related to her parents' separation and episodes of school bullying. Menarche had occurred 3 years earlier, but secondary amenorrhea had been present for 4 months. She denied vomiting, laxative use, or fear of gaining weight but admitted that she “felt better when eating less.”

Physical examination was unremarkable, but anxiety and a selective, unbalanced diet were noted, prompting referral for nutritional and psychological counseling.

Psychological assessment revealed perfectionism, anxiety, and body image distortion, raising suspicion of a restrictive eating disorder (ED). Given the presence of malnutrition and amenorrhea, an initial diagnosis of AN was made.

Despite dietary support, her weight continued to decline, accompanied by social withdrawal, anxiety, and persistent concern about food and body image. Subsequently, she experienced two febrile episodes: the first attributed to infectious mononucleosis and the second associated with erythema nodosum. The restrictive eating behavior progressively worsened, and she started to complain of mild abdominal pain.

She was therefore admitted for further investigations almost 8 months after symptom onset. She appeared severely undernourished (height 173 cm, >97th percentile; weight 38 kg, <3rd percentile; body mass index 13.7). Physical examination showed cachexia, dehydration, muscle wasting, and bilateral tender nodules on the lower limbs. Laboratory tests revealed leukocytosis, anemia, thrombocytosis, hypoalbuminaemia, and elevated C‐reactive protein and erythrocyte sedimentation rate.

Magnetic resonance enterography and ileocolonoscopy demonstrated segmental ileal inflammation with extensive mucosal involvement and deep ulcerations, consistent with CD. For induction of remission, the choice between Exclusive Enteral Nutrition (EEN) and the Crohn's Disease Exclusion Diet (CDED) was extensively discussed with the patient and her family. Although EEN would have ensured strict caloric control, both the patient and her parents expressed reluctance toward an exclusively liquid diet; therefore, CDED combined with Partial Enteral Nutrition (PEN) was initiated.

Given the severity of malnutrition, the patient was closely monitored for refeeding syndrome. Prophylactic thiamine was administered prior to dietary treatment, and serum electrolytes remained stable throughout refeeding. However, despite 4 weeks of nutritional therapy, clinical remission was not achieved. Owing to disease severity and persistent symptoms, anti‐tumor necrosis factor (TNF) therapy was subsequently initiated. After discussion with the patient's parents, Adalimumab was selected as monotherapy due to the patient's psychological distress and refusal of hospital‐based intravenous treatment, with better acceptance of a subcutaneous home‐based regimen. This resulted in marked clinical and nutritional improvement, with a weight gain of 18 kg over 4 months.

## 3 DISCUSSION

The present case illustrates the complex diagnostic trajectory often encountered in adolescent‐onset CD, initially manifesting with marked weight loss, amenorrhea, and emotional distress in the context of parental separation.

CD and AN share overlapping features, including weight loss, reduced oral intake, and restrictive eating patterns. In CD, postprandial abdominal pain and gastrointestinal discomfort may lead to food avoidance and restrictive consumption, thereby mimicking the behavioral features of AN. Clinicians observing such patterns may prematurely attribute symptoms to a primary ED without adequately investigating potential organic aetiologies.^4^, ^5^

Given this overlap, it is essential not only to fulfill all diagnostic criteria but also to exclude organic causes before establishing a psychiatric diagnosis to avoid DD. In this context, early use of non‐invasive investigations is crucial: in experienced hands, intestinal ultrasound represents a rapid, non‐invasive, and cost‐effective first‐line tool to screen for suspected IBD, alongside fecal calprotectin, particularly when the indication and timing for gastrointestinal endoscopy remain uncertain.

In some cases, meeting the clinical criteria for AN according to the Diagnostic and Statistical Manual of Mental Disorders, 5th edition (DSM‐5) may not be sufficient, as underlying somatic diseases can mimic the same presentation. In our case, not all diagnostic criteria for AN were fulfilled, as the patient did not exhibit an intense fear of gaining weight.^6^

Reports of CD initially misdiagnosed as ED are rare.^2^, ^3^, ^7^, ^8^ The literature describes adolescent females in whom IBD mimicked a primary ED, delaying appropriate treatment.^5^ It remains uncertain whether IBD predisposes to ED, whether ED precedes IBD, or whether the relationship is bidirectional.^3^ Bayle and Bouvard described a patient with coexisting CD and AN, highlighting the diagnostic challenges arising from overlapping clinical features.^9^ Regardless of causality, such comorbidity complicates both diagnosis and management, increasing the risk of treatment delay and poorer outcomes. In patients with CD and coexisting ED, induction therapy requires a highly tailored approach. Nutritional strategies should be discussed within a shared decision‐making process, with careful monitoring of compliance and RS. Likewise, in psychologically vulnerable adolescents, therapeutic decisions should also consider treatment acceptability. In this context, the mode of biologic administration and treatment setting may significantly influence adherence and overall treatment success.

## 4 CONCLUSIONS

Although based on a single patient and therefore limiting generalizability, this case highlights a clinically relevant diagnostic pitfall: CD can be mistaken for AN due to overlapping features and cognitive bias. It underscores the importance of maintaining a low diagnostic threshold for IBD in adolescents presenting with significant weight loss and restrictive eating behaviors, even in the presence of psychological distress. Greater awareness and early use of non‐invasive biomarkers, especially fecal calprotectin and intestinal ultrasound, could help reduce DD.

## CONFLICT OF INTEREST STATEMENT

The authors declare no conflict of interest.

## ETHICS STATEMENT

Informed patient consent from parents was obtained for publication of the case details anonymously.
